# Supplementary material for: Mechanism of ATP hydrolysis dependent rotation of bacterial ATP synthase
Source: Nat Commun. 2023 Jul 10;14:4090. doi: 10.1038/s41467-023-39742-5 (PMC10333338; doi:10.1038/s41467-023-39742-5)
Supplement: Supplementary file 1 — Supplementary Information [file 41467_2023_39742_MOESM1_ESM.pdf]

Supplementary Fig.1

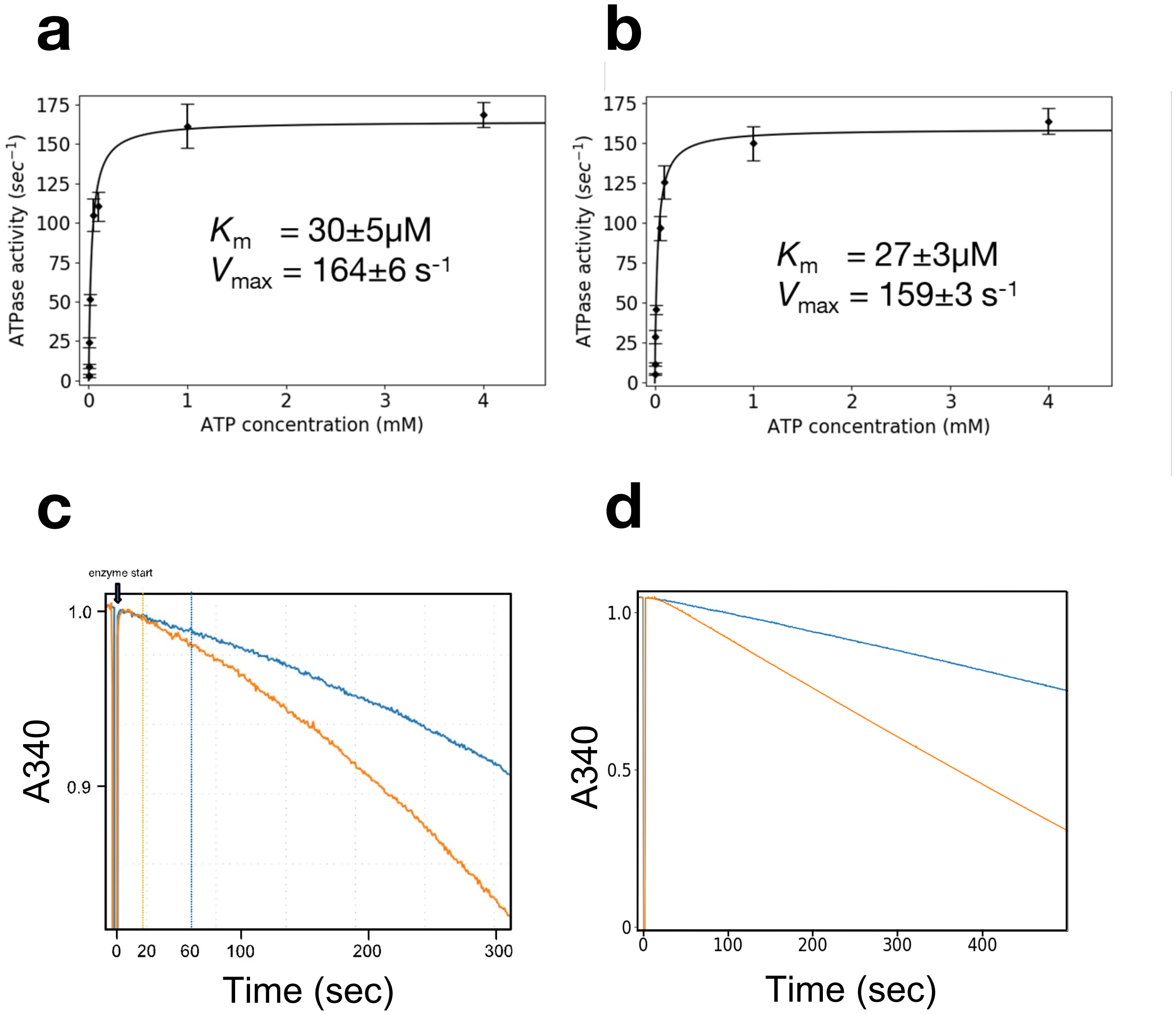

Supplementary Figure 1. Enzymatic properties of *ΔεCT*-F_oF_1. **a** Michaelis Menten curves showing the ATPase activity of *ΔεCT*-F_oF_1 with enzyme kinetics parameters of K_m = 30 μM and V_{max} = 164 s^{-1}. **b** Michaelis Menten curves showing the ATPase activity of nucleotide-depleted *ΔεCT*-F_oF_1 with enzyme kinetics parameters of K_m = 27μM and V_{max} = 159 s^{-1}. Three measurements were repeated at each concentration and the points are the average of the three experimental values. Error bars represent the standard deviation of ATPase activity from the mean. **c** Comparison of ATPase activity of *ΔεCT*-F_oF_1 at 25μM ATP (blue) and 26 mM ATP (orange). Measurements of ATPase activity were described in Methods section. Arrows represents the reaction time at low or high [ATP] condition, respectively. **d** ATPase activity of *ΔεCT*-F_oF_1 at 26 mM ATP-Mg, in the presence of 0.1% LDAO (orange) and without LDAO (blue).

Supplementary Fig.2

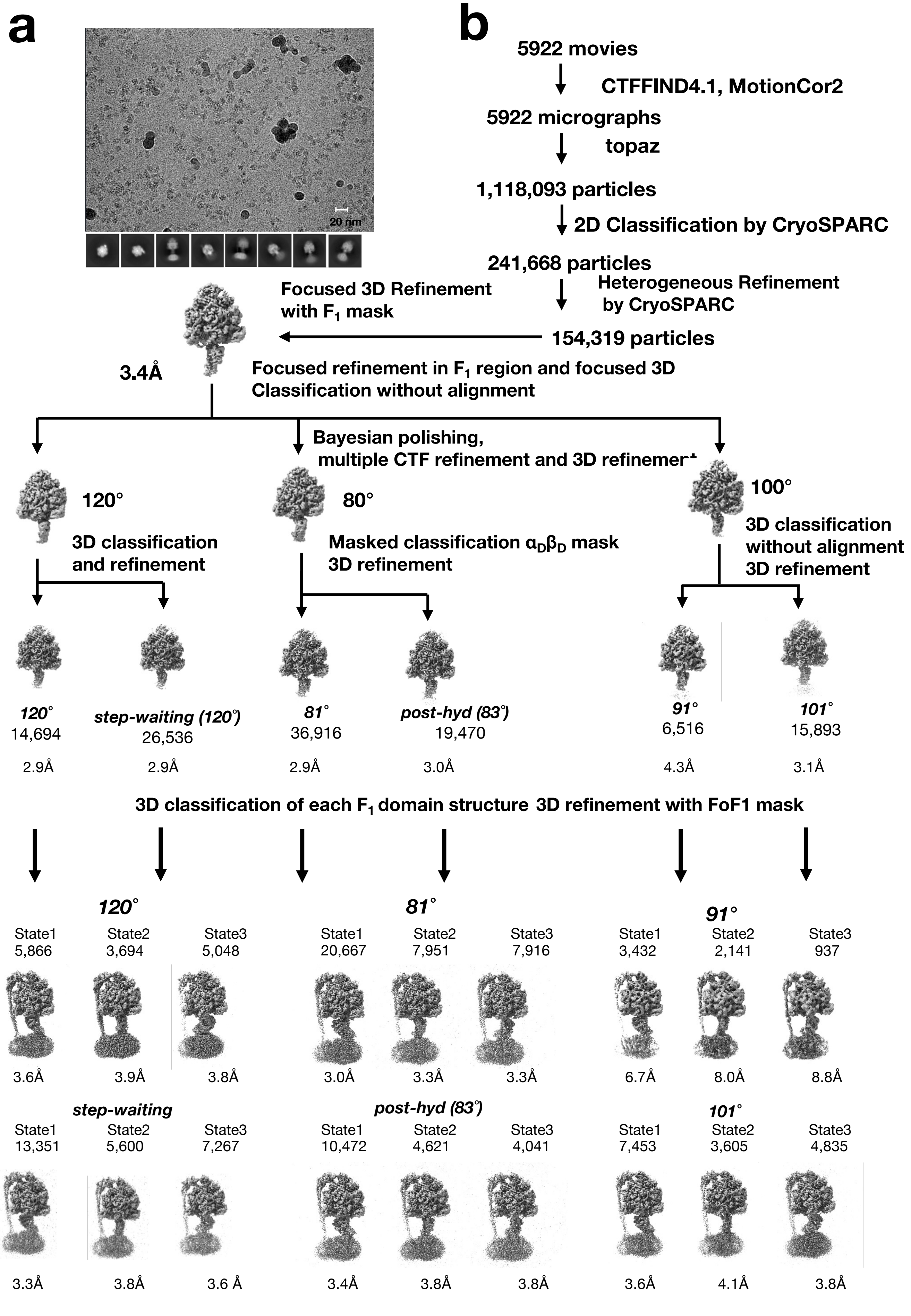

c

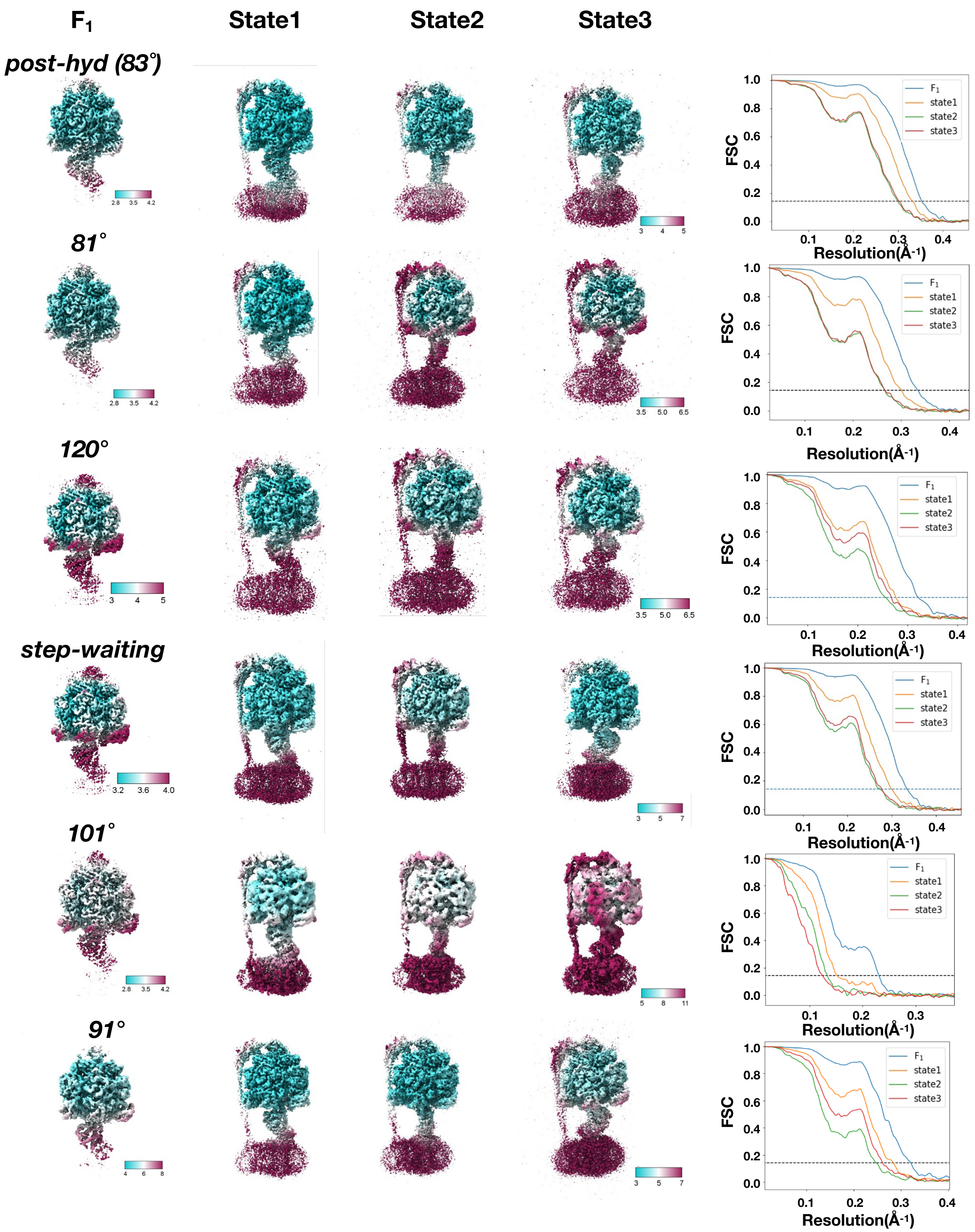

F_1
State1
State2
State3
post-hyd (83°)
81°
120°
step-waiting
101°
91°
FSC
Resolution(Å-1)
F1
state1
state2
state3

Supplementary Figure 2 Flow charts describing image acquisition and structural analysis allowing reconstitution of the 3D structures of *ΔεCT*-F_oF_1 at 6 mM [ATP]. **a** A typical micrograph of *ΔεCT*-F_oF_1 at 26 mM [ATP](upper) and 2D classes (lower). **b** Flow chart of single particle analysis for *ΔεCT*-F_oF_1 at 26 mM [ATP]. The selected 154k particles were subjected to focused refinement in the F_1 domain and focused 3D classification without alignment, resulting in three classes of 120°, 80°, and 100° rotation of the γ subunit. After 3D classification of the 120° structure, two subclasses were obtained, termed *120°* and *step-waiting*, respectively. *81°* and *post-hyd* (83°) were identified by $\alpha_D\beta_D$ masked classification of the 80° structure. From the particles of the 100° structure, *91°* and *101°* were obtained by 3D classification without alignment. The six intermediates (*81°, pot-hyd, 91°, 101°, 120°*, and *step-waiting*) were subjected to further classification with a F_oF_1 mask, resulting in three rotational states of six intermediates. **c** Resmaps of six intermediates of the three major rotational states. FSC curves for intermediates of F_1 domain and F_oF_1 are shown in the right hand panels.

Supplementary Fig.3

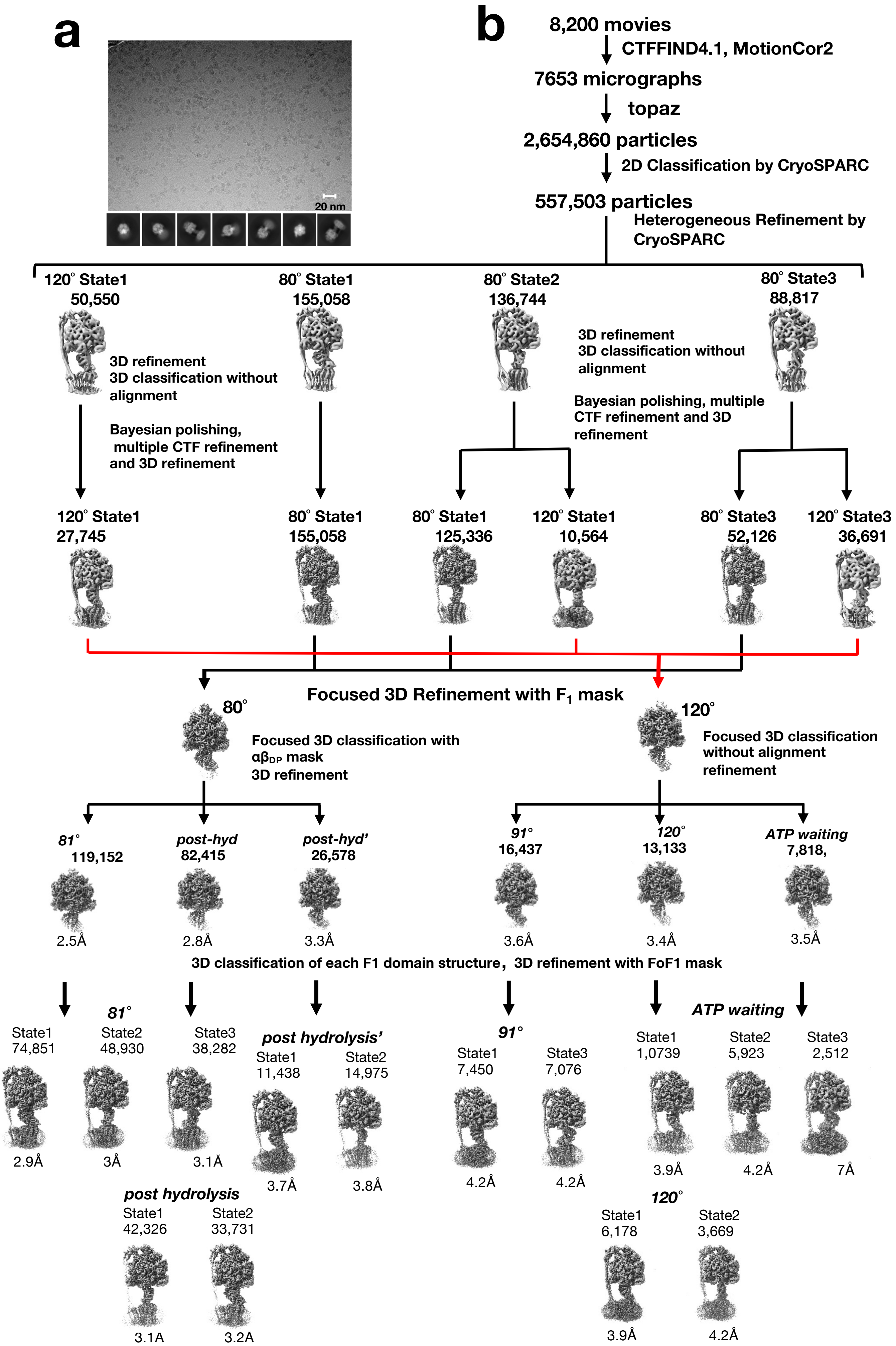

c

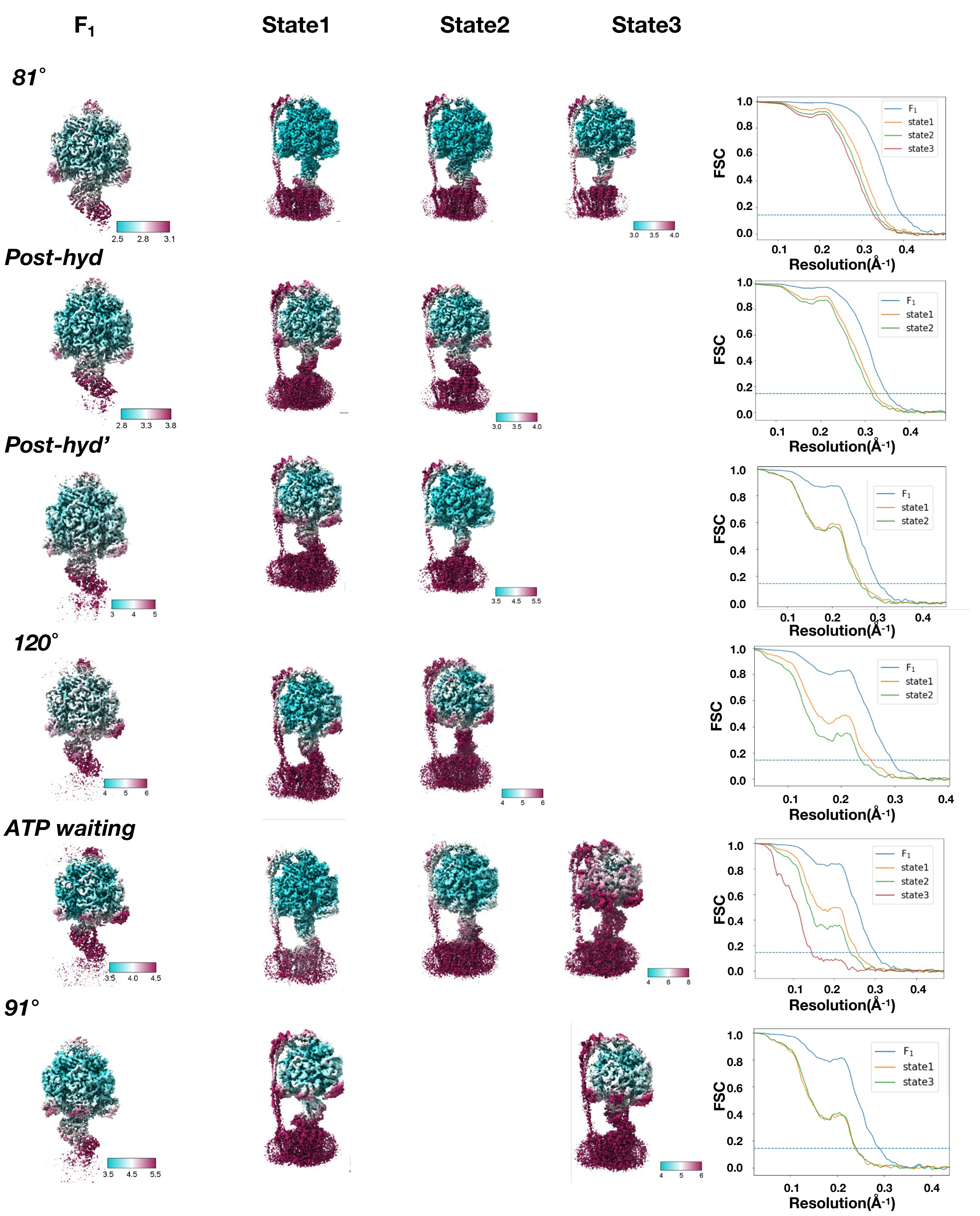

F1
State1
State2
State3
81°
Post-hyd
Post-hyd'
120°
ATP waiting
91°
FSC
Resolution(Å-1)
F1
state1
state2
state3

Supplementary Figure 3. Flow charts describing image acquisition and structural analysis allowing reconstitution of the 3D structures of *ΔεCT*-F_oF_1 at 25 μM [ATP]. **a** A typical micrograph of *ΔεCT*-F_oF_1 at 25 μM [ATP](upper) and the 2D classes (lower). **b** Flow chart of single particle analysis for *ΔεCT*-F_oF_1 at 25 μM [ATP]. The Selected 557 k particles were subjected to heterogeneous refinement using CryoSparc. The resulting three 80° structures and one 120° structure were further classified, resulting in three 120° structures and three 80° structures. The particles of 120° and 80° structures were individually combined. The particles of 80° structure were subjected to focused 3D classification using a $\alpha_D\beta_D$ mask, resulting in *81*° and two *post-hyd* structures. The particles for the 120° structure were subjected to focused 3D classification without alignment, resulting in *91*°, *120*°, and *ATP waiting*. The F_oF_1 particles of these six intermediates were subjected to further classification using a F_oF_1 mask, respectively. Two or three rotational states for six intermediates were classified, respectively. **c** Resmaps of six intermediates of two or three rotational states, respectively. The FSC curves for the intermediates of the F_1 domain and F_oF_1 are shown in the right hand panels.

Supplementary Fig.4

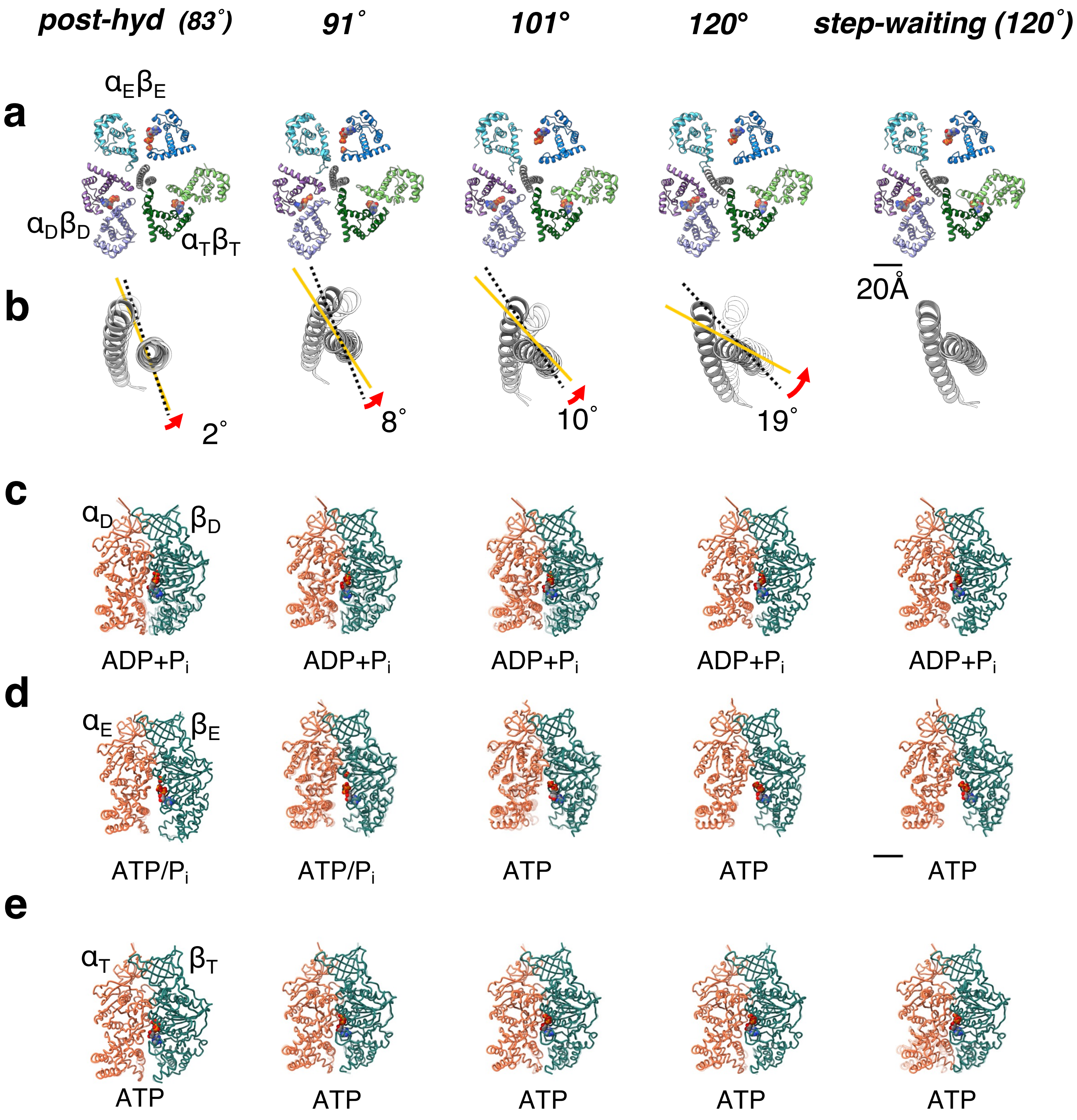

Supplementary Figure 4. Structure of 5 intermediates captured during the 40° step at high [ATP]. a Cross section of F_1 domain at the catalytic site. Each catalytic dimer is shown in ribbon representation and colored as detailed in Figure 1. The bound nucleotides to β subunits are represented as spheres. **b** Rotation angle of the γ subunit relative to that of the 0° structure. The structure of the γ subunit at the previous rotation angle is shown in white. **c-e** Structure of αβ dimers; $\alpha_D\beta_D$ (**c**), $\alpha_E\beta_E$ (**d**), $\alpha_T\beta_T$ (**e**). The bound nucleotide and Pi at the interface of each dimer is represented as spheres with the specific bound molecules labelled under the structures. The superimposed αβ dimers in previous angle are shown translucent, respectively.

Supplementary Fig.5

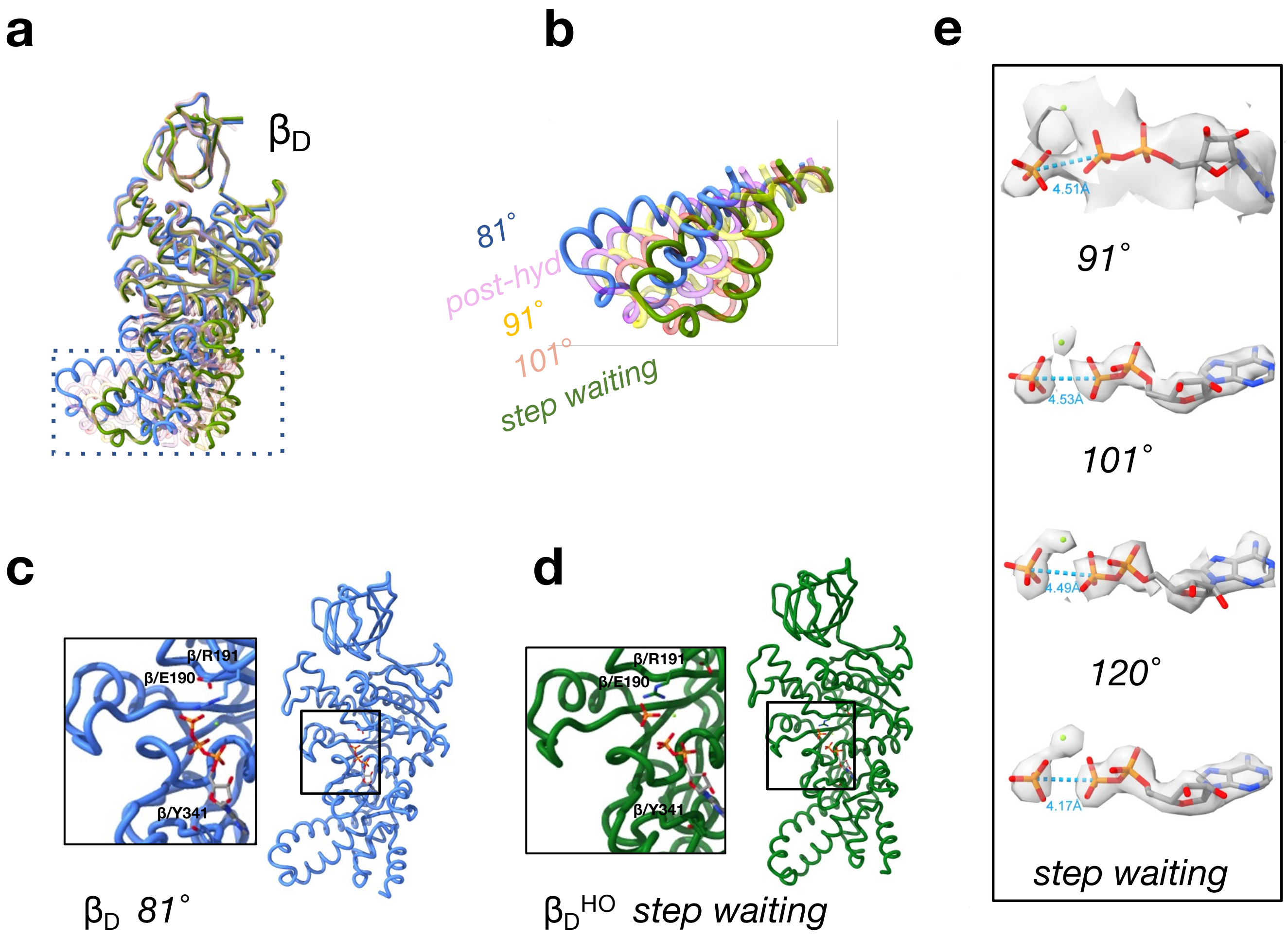

Supplementary Figure 5. Open motion of $\alpha_D\beta_D$ upon ATP hydrolysis. **a** superimposition of β subunit in *81°* with that in *post-hyd, 91°, 101°*, and *ATP waiting*. **b** motion of the *CT* loop helix domain in the conformation changes from *81°* to *step-waiting*. Structure of β_D in *81°* (**c**) and *step waiting structures* (**d**). The magnified views of the nucleotide binding site are shown in the left panels, respectively. ATP, ADP, Pi and coordinated amino acid residues are shown as sticks. **e** EM density of ATP/ADP+Pi bound to $\alpha_D\beta_D$, with the distance between β-phosphate and γ-phosphate or Pi indicated with a dotted line. Each nucleotide is represented by stick and magnesium ion by green sphere.

Supplementary Fig.6

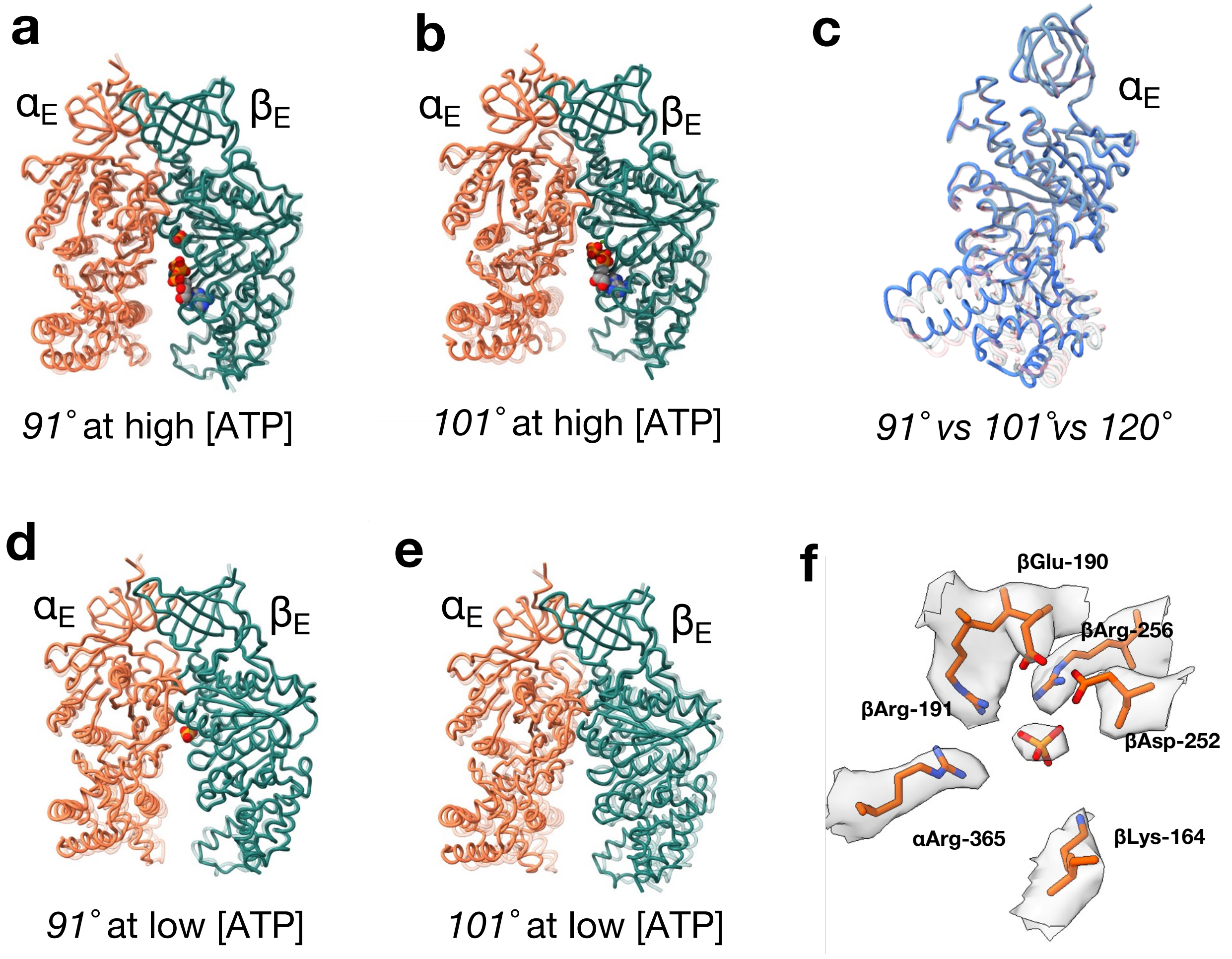

Supplementary Figure 6. Conformational changes of $\alpha_E\beta_E$ during the 40° rotation step. **a** Transparent $\alpha_E\beta_E$ in *post-hyd* is superimposed with $\alpha_E\beta_E$ in *91°* at high [ATP]. α_E and β_E are colored orange and green respectively. Bound ADP and Pi are represented by spheres. **b** Transparent $\alpha_E\beta_E$ in *91°* is superimposed with $\alpha_E\beta_E$ in *101°* at high [ATP]. **c** Superimposition of α_E in *120°(light blue chain)* with α_E in *101°(pink chain)* and α_E in *91°(transparent pink chain)*. **d** Transparent $\alpha_E\beta_E$ in *post-hyd* superimposed with $\alpha_E\beta_E$ in *91°* at low [ATP]. **e** Transparent $\alpha_E\beta_E$ in *91°* superimposed with $\alpha_E\beta_E$ in *101°* at low [ATP]. **f** Bound Pi and coordinated amino acid residues in $\alpha_E\beta_E$ of *post-hyd* with the density map.

Supplementary Fig.7

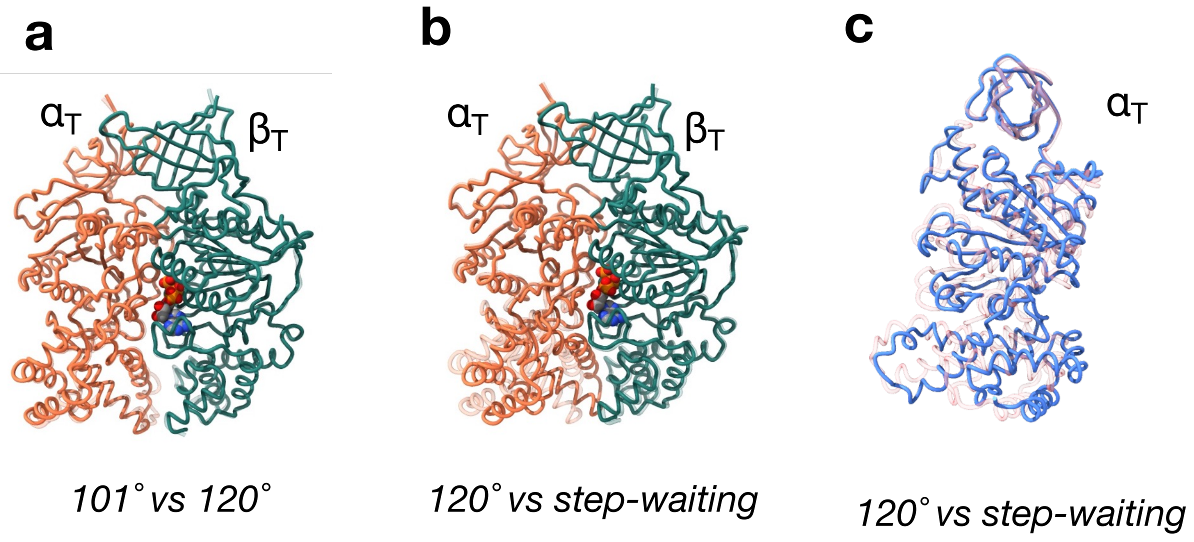

Supplementary Figure 7. Conformational changes of α_T between *120°* and *step-waiting*. **a** Transparent $\alpha_T\beta_T$ in *101°* is superimposed with $\alpha_T\beta_T$ in *120°*. The α and β subunits are colored and green, respectively. Bound ATP is represented by spheres. **b** Transparent $\alpha_T\beta_T$ in *120°* is superimposed with $\alpha_T\beta_T$ in *step-waiting*. **c** Superimposition of α_T subunit in *step-waiting (light blue chain)* with α subunit in *120° (transparent pink chain)*.

Supplementary Fig.8

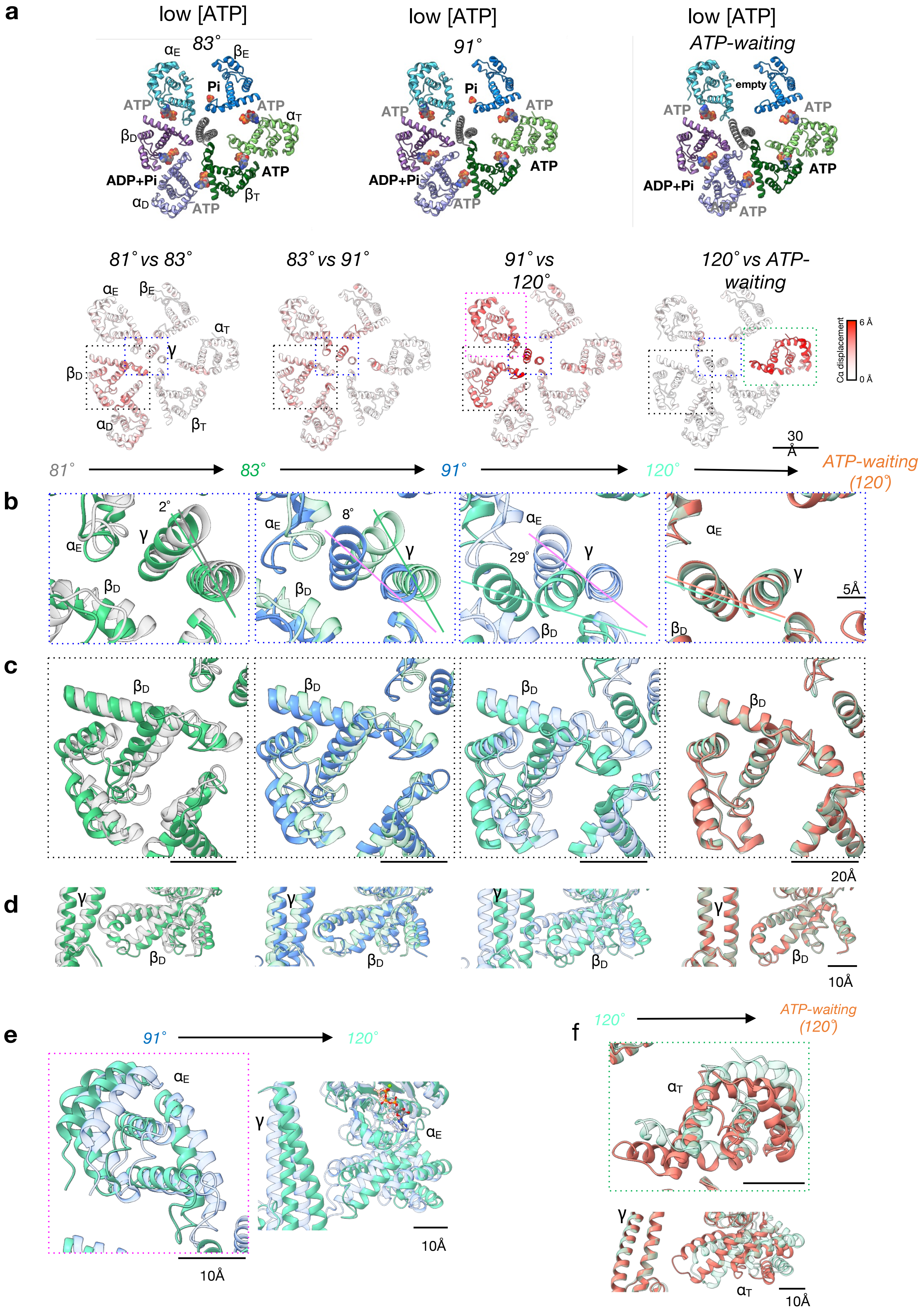

Supplementary Figure 8. Structure comparison of 5 intermediates captured during the 40° step at low [ATP]. **a** *upper,* Cross section of the F_1 domain showing nucleotide occupancy in the catalytic sites. *lower, 81°, post-hyd, 91°, 120°,* and *ATP-waiting* structures are arranged from left to right. The $C\alpha$ displacement relative to the next structure (*right side*) is indicated by the red-white color gradient. The dashed square indicates the area of this figure shown in the zoomed in view in **b-f**. **b-f** Comparison of each structure to the following one. **b** compares the γ subunit and its surroundings, and the different rotation angles between each γ subunit and that of the next structure are indicated by different colored lines. **c** shows a upper view of β_D, **d** compares β_D from a side view, Panels **e** and **f** compare to α_T and α_E, respectively. Each cartoon chain is colored as *81*°(grey), *83*°(green), *91*°(blue), *120*°(light green), and *ATP-waiting* (orange).

Supplementary Fig.9

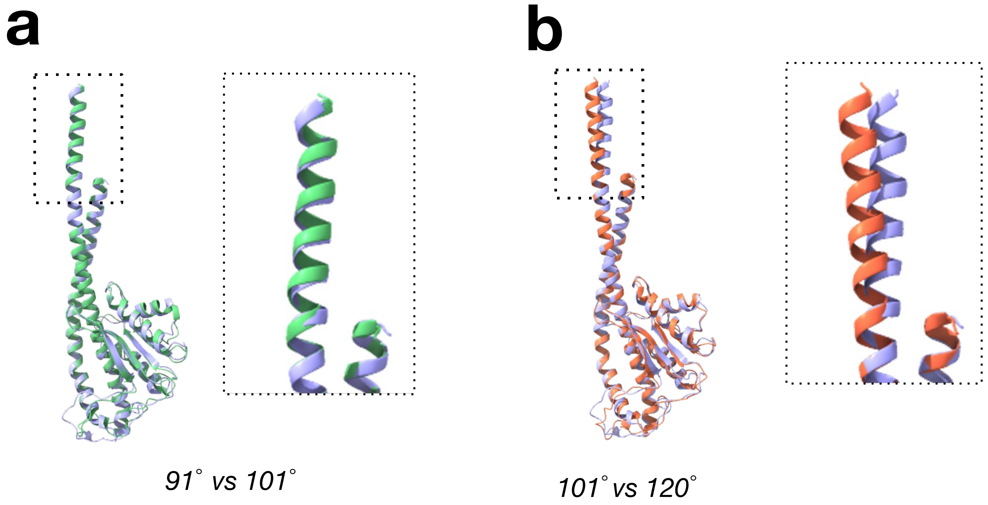

Supplementary Figure 9. Conformational changes of γ subunit between *91˚*(green) and *101˚*(blue) (**a**), and *101˚* and *120˚*(orange) (**b**). The magnified views of the C termini helices are shown in the right panels, respectively.

Supplementary Fig.10

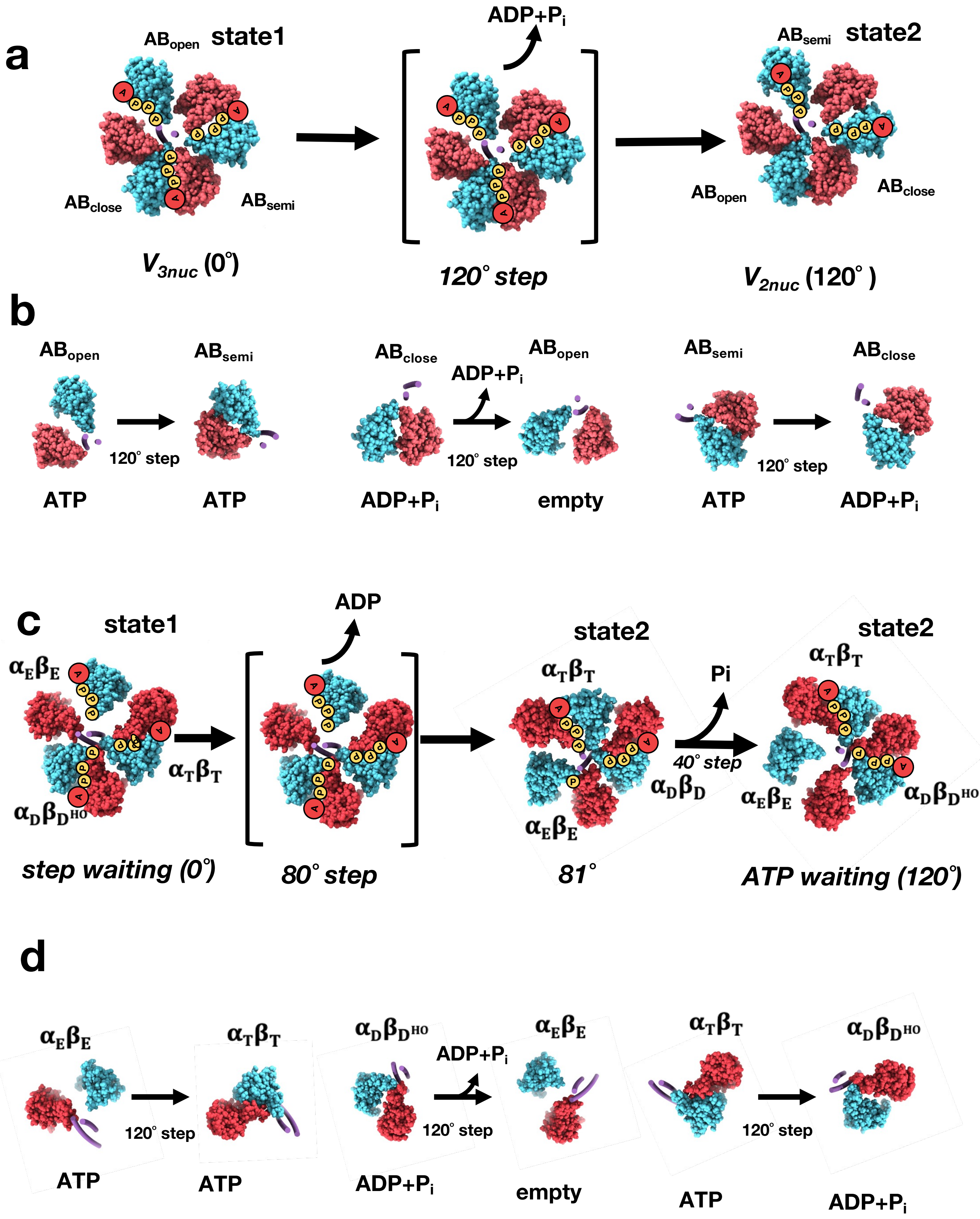

Supplementary Figure 10. Rotation schemes of rotary ATPases. **a** Structural change of the V_1 domain in V/A-ATPase during a complete 120° rotation. V_{3nuc}, generated by binding of ATP to V_{2nuc} in state 1, initiates the 120° rotation, resulting in V_{2nuc} in state 2. **b** Three catalytic events occur simultaneously at the three catalytic sites; closure of AB_{open} caused by binding of ATP, release of ADP and Pi from AB_{closed} by an opening motion of AB_{closed}, and hydrolysis of ATP in AB_{semi}, coupled with the 120° rotation of the central DF stalk. **c** Structural changes for F_1 domain in F_oF_1 during the 120° step. **d** During the 120° rotation of the γ subunit, three catalytic events also occur simultaneously at the three catalytic sites; closure of $\alpha_E\beta_E$ caused by binding of ATP, release of ADP and Pi from $\alpha_D\beta_D$ by an opening motion of $\alpha_D\beta_D$, and hydrolysis of ATP in $\alpha_T\beta_T$.

Supplementary Table 1 RMSD values for F_1 domain of each structure. The F_1 domains were superimposed on the β/10-80 a.a. and α/30-90 a.a., then the values for the backbone of F_1 domain were calculated using UCSF ChimeraX software.

low [ATP] \ high [ATP]	***81°***	***post-hyd***	***91°***	***101°***	***120°***	***step-waiting***
81°	0.79	1.25	2.00	2.71	2.77	2.71
post-hyd	1.01	1.08	1.85	2.43	2.48	3.22
91°	1.57	1.41	1.68	1.99	2.01	2.84
101°						
120°	2.69	2.55	2.36	1.38	0.77	1.82
ATP-waiting	3.25	3.14	2.79	1.72	1.53	0.66

Supplementary Table 2A Cryo-EM data collection, refinement and validation statistics for F_oF_1 at high [ATP]

	81°	*post-hyd*	*91°*	*101°*	*120°*	*step waiting*
EMDB ID	34748	34749	34750	34751	34752	34753
PDB ID	8HH1	8HH2	8HH3	8HH4	8HH5	8HH6
**Data collection and processing**						
Magnification	81,000	81,000	81,000	81,000	81,000	81,000
Voltage(kV)	300	300	300	300	300	300
Microscope	Titan Krios	Titan Krios	Titan Krios	Titan Krios	Titan Krios	Titan Krios
Total dose (e^-/Å^2)	60	60	60	60	60	60
Pixel size(Å/pix)	0.88	0.88	0.88	0.88	0.88	0.88
Defocus range(μm)	-0.8 to -2.0	-0.8 to -2.0	-0.8 to -2.0	-0.8 to -2.0	-0.8 to -2.0	-0.8 to -2.0
symmetry imposed	C1	C1	C1	C1	C1	C1
Initial particle	1,118,093	1,118,093	1,118,093	1,118,093	1,118,093	1,118,093
Final Particle	36,916	19,470	6,516	15,893	14,694	26,536
Map resolution(Å)	2.9	3.0	4.3	3.1	2.9	2.9
FSC threshold	0.143	0.143	0.143	0.143	0.143	0.143
**Refinement**						
Initial model used	This study	This study	This study	This study	This study	This study
Model resolution	2.9	3.1	4.3	3.3	3.2	3.2
FSC threshold	0.5	0.5	0.5	0.5	0.5	0.5
**Model composition**						
Nonhydrogen atoms	24280	24282	24288	24284	24284	24284
Protein residues	3129	3129	3129	3129	3129	3129
Ligands	5MG,6ATP, ,1PO_4	5MG,5ATP, 1ADP,2PO_4	5MG,5ATP, 1ADP,2PO_4	6MG,5ATP, 1ADP,1PO_4	6MG,5ATP, 1ADP,1PO_4	6MG,5ATP, 1ADP,1PO_4
**R.m.s deviations**						
Bond length (Å)	0.002	0.004	0.003	0.004	0.003	0.002
Bond Angles (°)	0.54	0.647	0.617	0.646	0.609	0.585
**Validation**						
MolProbity score	1.19	1.22	1.92	1.43	1.35	1.32
EMRinger score	3.96	3.2	1.03	3.01	3.47	3.28
Clashscore	4.1	4.06	10.3	4.12	5.62	4.98
Rotamer outlier (%)	0	0.51	0.12	0.04	0	0
CaBALM outlier (%)	1.61	1.84	2.87	2.26	1.68	1.84
**Ramachandran plot**						
Favored (%)	98.11	97.88	94.35	96.44	97.78	97.72
Allowed (%)	1.93	2.05	5.65	3.53	2.18	2.28
Disallowed (%)	0	0.06	0	0.03	0	0

Supplementary Table 2B Cryo-EM data collection, refinement and validation statistics for F_oF_1 at low [ATP]

	81°	*post-hyd*	*Post-hyd'*	*91°*	*120°*	*step waiting*
EMDB ID	34754	34755	34760	34756	34757	34758
PDB ID	8HH7	8HH8	8HHC	8HH9	8HHA	8HHB
**Data collection and processing**						
Magnification	81,000	81,000	81,000	81,000	81,000	81,000
Voltage(kV)	300	300	300	300	300	300
Microscope	Titan Krios	Titan Krios	Titan Krios	Titan Krios	Titan Krios	Titan Krios
Total dose (e^-/Å^2)	60	60	60	60	60	60
Pixel size(Å/pix)	0.88	0.88	0.88	0.88	0.88	0.88
Defocus range(µm)	-0.8 to -2.0	-0.8 to -2.0	-0.8 to -2.0	-0.8 to -2.0	-0.8 to -2.0	-0.8 to -2.0
symmetry imposed	C1	C1	C1	C1	C1	C1
Initial particle	2,654,860	2,654,860	2,654,860	2,654,860	2,654,860	2,654,860
Final Particle	119,152	82,415	26,578	16,437	13,133	7,818
Map resolution(Å)	2.5	2.8	3.3	3.6	3.4	3.5
FSC threshold	0.143	0.143	0.143	0.143	0.143	0.143
**Refinement**						
Initial model used	This study	This study	This study	This study	This study	This study
Model resolution	2.7	3	3.4	3.6	3.5	3.5
FSC threshold	0.5	0.5	0.5	0.5	0.5	0.5
**Model composition**						
Nonhydrogen atoms	24280	24282	24288	24284	24284	24284
Protein residues	3129	3129	3129	3129	3129	3129
Ligands	5MG,6ATP,	5MG,5ATP,	5MG,5ATP,	6MG,5ATP,	6MG,5ATP,	6MG,5ATP,
	,1PO_4	1ADP,2PO_4	1ADP,2PO_4	1ADP,1PO_4	1ADP,1PO_4	1ADP,1PO_4
**R.m.s deviations**						
Bond length (Å)	0.002	0.004	0.003	0.004	0.003	0.002
Bond Angles (°)	0.54	0.647	0.617	0.646	0.609	0.585
**Validation**						
MolProbity score	1.19	1.22	1.92	1.43	1.35	1.32
EMRinger score	3.96	3.2	1.03	3.01	3.47	3.28
Clashscore	4.1	4.06	10.3	4.12	5.62	4.98
Rotamer outlier (%)	0	0.51	0.12	0.04	0	0
CaBALM outlier (%)	1.61	1.84	2.87	2.26	1.68	1.84
**Ramachandran plot**						
Favored (%)	98.11	97.88	94.35	96.44	97.78	97.72
Allowed (%)	1.93	2.05	5.65	3.53	2.18	2.28
Disallowed (%)	0	0.06	0	0.03	0	0

Supplementary Table 2C Cryo-EM data collection, refinement for F_oF_1 at high [ATP]

	81°			***post-hyd***			***91°***		
	state1	state2	state3	state1	state2	state3	state1	state2	state3
EMDB ID	34759	34761	34763	34762	34764	34765	34766	34767	35373
**Data collection and processing**									
Magnification	81,000	81,000	81,000	81,000	81,000	81,000	81,000	81,000	81,000
Microscope	Titan Krios	Titan Krios	Titan Krios	Titan Krios	Titan Krios	Titan Krios	Titan Krios	Titan Krios	Titan Krios
Voltage(kV)	300	300	300	300	300	300	300	300	300
Total dose (e^-/Å^2)	60	60	60	60	60	60	60	60	60
Pixel size(Å/pix)	0.88	0.88	0.88	0.88	0.88	0.88	0.88	0.88	0.88
Defocus range(μm)	-0.8 to -2.0	-0.8 to -2.0	-0.8 to -2.0	-0.8 to -2.0	-0.8 to -2.0	-0.8 to -2.0	-0.8 to -2.0	-0.8 to -2.0	-0.8 to -2.0
symmetry imposed	C1	C1	C1	C1	C1	C1	C1	C1	C1
Initial particle	1,381,269	1,381,269	1,381,269	1,381,269	1,381,269	1,381,269	1,381,269	1,381,269	1,381,269
Final Particle	20,667	7,951	7,916	10,472	4,621	4,041	3,432	2,141	937
Map resolution(Å)	3.0	3.3	3.3	3.4	3.8	3.8	6.7	8.0	8.8
FSC threshold	0.143	0.143	0.143	0.143	0.143	0.143	0.143	0.143	0.143

	101°			***120°***			***step waiting***		
	state1	state2	state3	state1	state2	state3	state1	state2	state3
EMDB ID	34768	34769	34770	34771	34772	34773	34774	34775	34776
**Data collection and processing**									
Magnification	81,000	81,000	81,000	81,000	81,000	81,000	81,000	81,000	81,000
Microscope	Titan Krios	Titan Krios	Titan Krios	Titan Krios	Titan Krios	Titan Krios	Titan Krios	Titan Krios	Titan Krios
Voltage(kV)	300	300	300	300	300	300	300	300	300
Total dose (e^-/Å^2)	60	60	60	60	60	60	60	60	60
Pixel size(Å/pix)	0.88	0.88	0.88	0.88	0.88	0.88	0.88	0.88	0.88
Defocus range(μm)	-0.8 to -2.0	-0.8 to -2.0	-0.8 to -2.0	-0.8 to -2.0	-0.8 to -2.0	-0.8 to -2.0	-0.8 to -2.0	-0.8 to -2.0	-0.8 to -2.0
symmetry imposed	C1	C1	C1	C1	C1	C1	C1	C1	C1
Initial particle	1,381,269	1,381,269	1,381,269	1,381,269	1,381,269	1,381,269	1,381,269	1,381,269	1,381,269
Final Particle	7,453	3,605	4,835	5,866	3,694	5,048	13,351	5,600	7,267
Map resolution(Å)	3.6	4.1	3.8	3.6	3.9	3.8	3.3	3.8	3.6
FSC threshold	0.143	0.143	0.143	0.143	0.143	0.143	0.143	0.143	0.143

Supplementary Table 2D Cryo-EM data collection, refinement for F_oF_1 at low [ATP]

	81°			***post-hyd***		***post-hyd'***	
	state1	state2	state3	state1	state2	state1	state2
EMDB ID	34777	34779	34780	34778	34781	34782	23783
**Data collection and processing**							
Magnification	81,000	81,000	81,000	81,000	81,000	81,000	81,000
Microscope	Titan Krios	Titan Krios	Titan Krios	Titan Krios	Titan Krios	Titan Krios	Titan Krios
oltage(kV)	300	300	300	300	300	300	300
Total dose (e^-/Å^2)	60	60	60	60	60	60	60
Pixel size(Å/pix)	0.88	0.88	0.88	0.88	0.88	0.88	0.88
Defocus range(μm)	-0.8 to -2.0	-0.8 to -2.0	-0.8 to -2.0	-0.8 to -2.0	-0.8 to -2.0	-0.8 to -2.0	-0.8 to -2.0
symmetry imposed	C1	C1	C1	C1	C1	C1	C1
Initial particle	2,654,860	2,654,860	2,654,860	2,654,860	2,654,860	2,654,860	2,654,860
Final Particle	74,851	48, 930	38,282	42,326	33,731	11,438	14,975
Map resolution(Å)	2.9	3.0	3.1	3.1	3.2	3.7	3.8
FSC threshold	0.143	0.143	0.143	0.143	0.143	0.143	0.143

	91°		***120°***		***step waiting***		
	state1	state3	state1	state2	state1	state2	state3
EMDB ID	34784	34785	34786	34787	34788	34,789	34,790
**Data collection and processing**							
Magnification	81,000	81,000	81,000	81,000	81,000	81,000	81,000
Microscope	Titan Krios	Titan Krios	Titan Krios	Titan Krios	Titan Krios	Titan Krios	Titan Krios
Voltage(kV)	300	300	300	300	300	300	300
Total dose (e^-/Å^2)	60	60	60	60	60	60	60
Pixel size(Å/pix)	0.88	0.88	0.88	0.88	0.88	0.88	0.88
Defocus range(μm)	-0.8 to -2.0	-0.8 to -2.0	-0.8 to -2.0	-0.8 to -2.0	-0.8 to -2.0	-0.8 to -2.0	-0.8 to -2.0
symmetry imposed	C1	C1	C1	C1	C1	C1	C1
Initial particle	2,654,860	2,654,860	2,654,860	2,654,860	2,654,860	2,654,860	2,654,860
Final Particle	7,450	7,076	6,178	3,669	10,739	5,923	2,512
Map resolution(Å)	4.2	4.2	3.9	4.2	3.9	4.2	7.0
FSC threshold	0.143	0.143	0.143	0.143	0.143	0.143	0.143
