# Supplementary material for: Mechanism of ATP hydrolysis dependent rotation of bacterial ATP synthase
Source: Nat Commun. 2023 Jul 10;14:4090. doi: 10.1038/s41467-023-39742-5 (PMC10333338; doi:10.1038/s41467-023-39742-5)
Supplement: Supplementary file 3 — Description to Additional Supplementary Information [file 41467_2023_39742_MOESM3_ESM.pdf]

Description of Additional Supplementary Files

Supplementary movie 1. Structural changes in F_1 domain during 120˚ step.
